# Does the non-absorbable suture closure of the jejunal mesenteric defect reduce the incidence and severity of internal hernias after laparoscopic Roux-en-Y gastric bypass?

**DOI:** 10.1007/s00423-021-02180-2

**Published:** 2021-05-22

**Authors:** Romano Schneider, Michaela Schulenburg, Marko Kraljević, Jennifer M. Klasen, Thomas Peters, Bettina Wölnerhanssen, Ralph Peterli

**Affiliations:** 1 Clarunis, Department of Visceral Surgery, University Center for Gastrointestinal and Liver Diseases, St. Claraspital and University Hospital Basel, CH-4002 Basel, Switzerland; 2 Interdisciplinary Center of Nutritional and Metabolic Diseases, St. Claraspital, CH-4058 Basel, Switzerland; 3 St. Clara Research Ltd, St. Claraspital, CH-4002 Basel, Switzerland

**Keywords:** Gastric bypass, Bariatric surgery, Internal hernia, Mesenteric defects

## Abstract

**Purpose:**

Internal hernias (IH) are frequent complications after laparoscopic Roux-en-Y gastric bypass (LRYGB). Closure of the jejunal mesenteric and the Petersen defect reduces IH incidence in prospective and retrospective trials. This study investigates whether closing the jejunal mesenteric space alone by non-absorbable suture and splitting the omentum can be beneficial to prevent IH after LRYGB.

**Methods:**

Observational cohort study of 785 patients undergoing linear LRYGB including omental split at a single institution, with 493 patients without jejunal mesenteric defect closure and 292 patients with closure by non-absorbable suture, and a minimal follow-up of 2 years. Patients were assessed for appearance and severity of IH. Additionally, open mesenteric gaps without herniated bowel as well as early obstructions due to kinking of the entero-enterostomy (EE) were explored.

**Results:**

Through primary mesenteric defect closure, the rate of manifest jejunal mesenteric and Petersen IH could be reduced from 6.5 to 3.8%, but without reaching statistical significance. The most common location for an IH was the jejunal mesenteric space, where defect closure during primary surgery reduced the rate of IH from 5.3 to 2.4%. Higher weight loss seemed to increase the risk of developing an IH.

**Conclusion:**

The closure of the jejunal mesenteric defect by non-absorbable suture may reduce the rate of IH at the jejunal mesenteric space after LRYGB. However, the beneficial effect in our collective is smaller than expected, particularly in patients with good weight loss. The Petersen IH rate remained low by consequent T-shape split of the omentum without suturing of the defect.

## Introduction

Metabolic surgery is a safe and efficient treatment for morbid obesity in terms of weight loss and amelioration of related comorbidities [1–5]. The laparoscopic Roux-en-Y gastric bypass (LRYGB) is a standard procedure worldwide [6–9]. Due to the growing number of patients undergoing LRYGB, surgeons are faced with increasing numbers of postoperative complications such as internal hernias (IH) [10–12]. In prospective and retrospective studies, closure of mesenteric defects reduces IH incidence after LRYGB with an antecolic alimentary limb (AL) [13, 14]. But adverse effects associated with closure of mesenteric defects, such as bowel obstruction by kinking of the entero-enterostomy (EE) or mesenteric hematomas, are infrequently seen [15]. Closing mesenteric defects is widely accepted, but the applied technique (e.g., metal clips or non-absorbable sutures) is still subject for investigation [16–18]. In addition to the closure technique, there also is a question as to whether closure of the jejunal mesenteric space (space between the EE and the mesentery) alone is sufficient. A large retrospective series strongly supports the recommendation to close the jejunal mesenteric defect and the Petersen space (between the AL and the transverse mesocolon) [19]. Also, in a meta-analysis performed by Geubbels et al., the combination of jejunal mesenteric space and Petersen space closure seems to generate the lowest rates of IH in the long-term [10].

In the past, we observed a reduction of IH from 12.3 to 5.8% by changing from circular stapled gastroenterostomy (GE) (biliopancreatic limb [BPL] oriented to the right) to linear stapled GE (BPL oriented to the left). In the original version of the linear stapled LRYGB technique described by Hans Lönroth, the mesenteric defects were not closed, but the omentum was routinely split in the vertical direction to facilitate a tension free anastomosis between AL and the pouch [7]. As a consequence of the evidence provided by Aghajani et al. and Stenberg et al., in 2016, we started closing mesenteric defects as part of our antecolic linear LRYGB technique [13, 17, 20]. However, though the rate of Petersen hernias was low in our previously published results, the new closure technique could cause obstruction of the transverse colon or bleeding at the transverse mesocolon. The aforementioned authors stopped splitting the omentum routinely and/or performed an incision of the mesentery to allow the EE to drop below the level of the transverse colon to prevent a Petersen hernia; we only closed the jejunal mesenteric defect by a non-absorbable suture and split the omentum in a T-shape. The objective of this study is to analyze possible benefits of suture closure of the jejunal mesenteric defect and simultaneous omentum T-shape split without suture of the Petersen space. In comparison to a group of patients undergoing LRYGB without closure of any defects, we analyzed the appearance, localization, and severity of IH.

## Materials and methods

This is an observational cohort study with prospectively collected data. Informed consent was obtained from all participants as a mandatory part of quality control in our hospital. The local ethics committee approved the study (EKNZ 2018/00356).

### Patients and data

At our institution, we perform approximately 300 bariatric operations per year. All patients with morbid obesity are assessed by an interdisciplinary team (endocrinologist, psychiatrist, nutritionist, and bariatric surgeon) in accordance with national guidelines. Before 2011, criteria to perform metabolic surgery in Switzerland included a body mass index (BMI) > 40 kg/m^2^ or > 35 kg/m^2^ with the presence of at least one comorbidity and failed conservative treatment over 2 years. From 2011 to the present, a BMI > 35–50 kg/m^2^ and failed conservative treatment over 2 years or 1 year with a BMI > 50 kg/m^2^ became preconditions to qualify for surgery [21]. All data, including demographic information, weight loss as well as complications and comorbidities are, prospectively entered into our clinical database.

In the context of our study, only primary LRYGB procedures and cases, which passed the 2-year follow-up period were included for analysis; patients who did not attend two consecutive standard appointments in our outpatient clinic were considered lost to follow-up.

### Surgical procedure

In 2009, we changed from the circular to the linear stapling LRYGB technique. Our technique has been described in detail previously [20]. In brief, the GE between the gastric pouch and the alimentary limb is created by two-thirds of the length of a 45-mm linear stapler magazine. The omentum is divided into a “T-shape” fashion (first widely separated parallel from the transverse colon and then split vertically), creating enough space for the antecolic AL to be placed without torsion and facilitating adhesions to potentially prevent bowel herniation. The 50-cm BPL was placed in the left side of the abdominal cavity, and the AL was orientated to the right. The creation of the EE was conducted using a 45-mm linear stapler side-to-side. A small window was created to allow the passage of the stapling device to divide the small intestine between the AL and the BPL close to the GE. When the jejunal mesenteric space was closed, we placed a non-absorbable barbed purse string-suture to close the vertex of the jejunal mesenteric space and closed the cranial part of the gap by running suture with the same thread.

### Internal hernia assessment

Included in the analysis were patients who received surgery for abdominal pain and/or signs of intestinal obstruction with a clinically high suspicion of IH. IH was defined as herniation of bowel through the jejunal mesenteric or the Petersen space detected in laparoscopy or laparotomy. Appearance of obstruction or ischemia was recorded separately. Obstruction was described as any obvious dilatation of the intestine, while ischemia was defined as bluish change of intestine color or venous congestion. In cases of ischemia, the need for bowel resection was included in the evaluation. Next to IH, open mesenteric gaps without content as well as early obstructions due to kinking of the EE and 30 days of complications were assessed separately.

### Statistical analysis

Continuous data were summarized using mean and standard deviation (SD). Categorical variables were summarized using counts and percentages. BMI change between study groups was compared by using an independent *t*-test. Time-to-event data were displayed using Kaplan-Meier survival curves with corresponding Log-rank (Mantel-Cox) tests. Numbers of active patients under surveillance were displayed as number at risk for different timepoints. Only the time-to-first event was considered due to very rare second events. Comparisons between study groups were reported showing hazard ratios (HR) with corresponding 95% confidence intervals (CI) and *p* values. All analyses were performed using GraphPad Prism 8 for Windows Version 8.4.1 [22].

## Results

### Patient characteristics

**Fig. 1:**
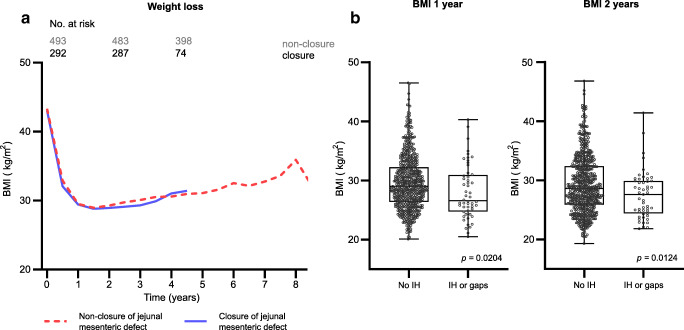
**a** Weight evolution curve showing weight in BMI, distinguishing patients with non-closure of the jejunal mesenteric defect from patients operated with closure of the jejunal mesenteric defect. Number of patients at risk (active patients) displayed on top. **b** BMI 1 and 2 years after primary LRYGB, distinguishing between the whole collective with no IH or no open mesenteric gap vs. all patients with IH or open mesenteric gap event. The boxes show the median and IQR. Single data points represent each individual patient, and the whiskers represent the minimum and maximum range of values. *P*-values displayed from unpaired *t*-test. BMI body mass index, SD standard deviation, IH internal hernia. Gaps: open mesenteric gaps at the jejunal mesenteric space or the Petersen space

In the period from 2/2009 to 3/2018, 820 patients underwent primary laparoscopic linear LRYGB at our institution. Of the 820 patients, 785 completed 2 years follow-up (95.7%). Of those 785 patients, the first 493 patients did not receive a closure of the jejunal mesenteric defect (non-closure group) and the remaining 292 patients did receive closure of the primary jejunal mesenteric defect (closure group). There was no statistically significant difference in baseline demographic data between the two groups (Table 2). Since we started closing the jejunal mesenteric defect in 2016, the non-closure group had a significantly longer follow-up time compared to the closure group (5.8 ± 2.1 years vs. 2.9 ± 0.8 years [*p* = 0.0001]). There was no statistically significant difference in weight loss between the groups at all time points (Table 1; Fig. 1)

**Table 1:** Patient characteristics, demographic data, follow-up, and weight evolution after LRYGB comparing the group with non-closure of the jejunal mesenteric defect and the group with closure of the jejunal mesenteric defect. Values are expressed as means ± standard deviation, and *p*-values are displayed from unpaired *t*-test. SD = standard deviation. BMI = body mass index. LRYGB = laparoscopic Roux-en-Y gastric bypass

	Non-closure of jejunal mesenteric defect	Closure of jejunal mesenteric defect	*p*
Patients (*n*)	493	292	
Age primary surgery (years ± SD)	41.83 ± 12.81	41.29 ± 12.45	0.9267
BMI primary surgery (kg/m^2^ ± SD)	43.32 ± 5.15	43.03 ± 5.13	0.4490
Female (*n* [%])	364 (73.83%)	218 (74.66%)	0.8661
Diabetes primary surgery (*n* [%]) -With Insulin (*n* [%])	83 (16.83%) 31 (6.29%)	43 (14.72%) 21 (7.19%)	0.4818 0.6571
Follow-up time (years ± SD)	5.75 ± 2.12	2.88 ± 0.79	<0.0001
BMI 1 year (kg/m^2^ ± SD)	29.51 ± 4.62	29.44 ± 4.47	0.8621
BMI 2 years (kg/m^2^ ± SD)	29.28 ± 4.84	28.94 ± 4.67	0.3655

### Early obstructions and complications of initial bariatric procedure

We observed 5 (1.0%) early obstructions in the non-closure group vs. two [0.7%] in the closed group (*p* ≥ 0.99). All early obstructions were seen within the first 2 weeks after surgery. Treatment consisted of bypassing the EE by side-side anastomosis between the end of the AL with the common limb (CL) and the end of the BPL with the CL in addition to the placement of a gastrostomy tube.

### Internal hernias

In total, 67 patients presented typical symptoms of an IH and received diagnostic laparoscopy (51 [10.3%] in the non-closure vs. 16 patients [5.5%] in the closure group [*p* = 0.02]). The average time from primary to revisional surgery was longer in the non-closure group than in the closure group (2.8 ± 2.2 years vs. 1.3 ± 1.1 years [*p* = 0.009]). BMI at the time of revisional surgery showed no difference between the groups. In both collectives, revisional surgery could be carried out laparoscopically in most cases (80.4% vs. 93.8% [*p* = 0.27]) (Table 2).

**Table 2:** Revisional surgery data and findings in exploratory laparoscopy/laparotomy comparing the group with non-closure of the jejunal mesenteric defect and the group with closure of the jejunal mesenteric defect. Values are expressed as counts and percentages or means ± standard deviation, and *p*-values are displayed from Fisher’s exact test or unpaired *t*-test. SD = standard deviation, BMI = body mass index, IH = internal hernia, LRYGB = laparoscopic Roux-en-Y gastric bypass

	Non-closure of jejunal mesenteric defect	Closure of jejunal mesenteric defect	*p*
Patients (*n*)	493	292	
Surgery with suspicion of IH (*n* [%])	51 (10.34%)	16 (5.48%)	0.0177*
Time to revisional surgery (years ± SD)	2.76 ± 2.15	1.29 ± 1.05	0.0090*
BMI at revisional surgery (kg/m^2^ ± SD)	28.06 ± 5.42	27.51 ± 3.76	0.7047
Revisional surgery carried out laparoscopically (*n* [%])	41 (80.39%)	15 (93.75%)	0.2745
Manifest IH (*n* [%])	32 (6.49%)	11 (3.77%)	0.1432
IH site			
Jejunal mesenteric (*n* [%])	26 (5.27%)	7 (2.40%)	0.0648
Petersen (*n* [%])	11 (2.23%)	4 (1.37%)	0.5908
Obstruction (*n* [%])	13 (2.64%)	5 (2.05%)	0.8108
Ischemia (*n* [%])	8 (1.62%)	2 (0.68%)	0.3369
Open gap jejunal mesenteric space (*n* [%])	25 (5.07%)	4 (1.37%)	0.0096*
Open gap Petersen space (*n* [%])	24 (4.87%)	11 (3.77%)	0.5921
30-day complications of primary LRYGB			
Early obstruction at EE (*n* [%])	5 (1.01%)	2 (0.68%)	> 0.9999
Leakage (*n* [%])	3 (0.61%)	1 (0.34%)	> 0.9999
Bleeding (*n* [%])	0 (0.00%)	1 (0.34%)	0.3720

The rate of overall manifest IH confirmed upon revisional laparoscopy could be reduced from 6.5 to 3.8% by primarily closing the jejunal mesenteric defect, but without reaching statistical significance (*p* = 0.14). The most common location for an IH was the jejunal mesenteric space, where defect closure during primary surgery could reduce the rate of IH from 5.3 to 2.4% (*p* = 0.06). The Petersen space hernia incidence was comparable with 2.2% in the non-closure vs. 1.4% in the closure group (*p* = 0.59). The only statistically significant difference that could be shown in absolute numbers was the reduction of an open jejunal mesenteric space without herniated bowel between the non-closure compared vs. the closure group (5.1% to 1.4% [*p* = 0.01]) (Table 2). In general, patients undergoing revisional surgery with suspicion of IH had lower BMI than patients without an event (28.1 ± 4.6 kg/m^2^ vs. 29.6 ± 4.5 kg/m^2^ [*p* = 0.02] after 1 year and 27.6 ± 4.1 kg/m^2^ vs. 29.3 ± 4.8 kg/m^2^ [*p* = 0.01] after 2 years, respectively). The prevalence of type 2 diabetes mellitus (T2DM) at baseline in patients undergoing revisional surgery with suspicion of IH was 11.7% in the non-closure vs. 12.5% in the closure group. Alternatively, 17.4% of patients without an event in the non-closure group and 14.9% of patients without an event in the closure group were suffering from T2DM at baseline.

**Fig. 2:**
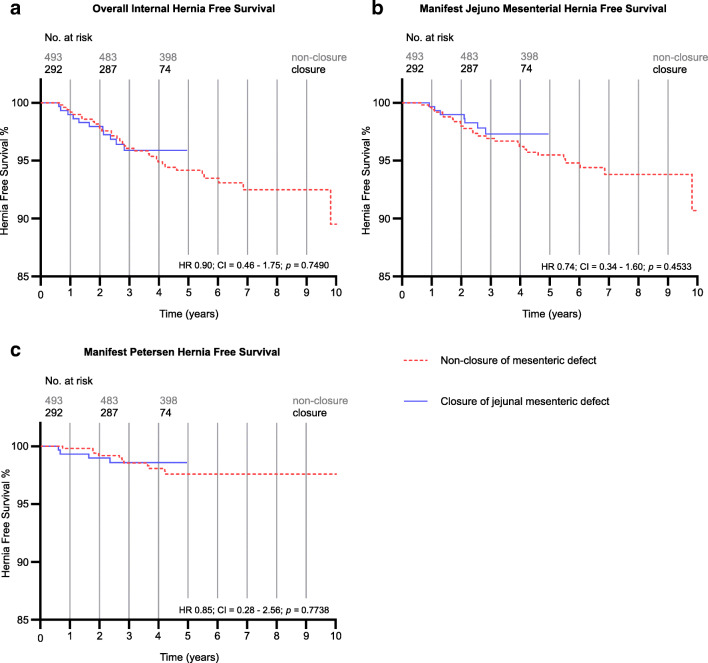
**a** Kaplan-Meyer survival curve showing all IH cases (i.e., all cases with herniated bowel). **b** IH cases at the jejunal mesenteric space. **c** IH cases at the Petersen space, respectively. Number of patients at risk (active patients) displayed on top. HR, 95% CI and *p*-values displayed from Log-rank (Mantel-Cox) test. HR hazard ratio, CI confidence interval, IH internal hernia

The IH and event-free survival figures demonstrate the appearance of IH or open mesenteric gaps over time. There was only a small benefit in overall manifest IH reduction (HR 0.90; CI = 0.46–1.75; *p* = 0.75) (Fig. 2a), manifest jejunal mesenteric hernias reduction (HR 0.74; CI = 0.34–1.60; *p =* 0.45) (Fig. 2b), and in hernias at the Petersen space (HR 0.85; CI = 0.28–2.56; *p =* 0.77) (Fig. 2c), for suture closure of the jejunal mesenteric defect. However, no IH occurred after 3 years in the closure group with 177 patients remaining at risk (Fig. 2)

In all patients undergoing revisional surgery, both gaps were closed with a non-absorbable running suture. Two patients with IH were pregnant (35 and 33 weeks of pregnancy, respectively). The patient in the 35th week underwent cesarean section and IH repair at the same time. Seven patients were managed and operated outside of our clinic. Only one patient who was operated externally needed small bowel resection (resection and reconstruction of the EE) due to severe ischemia caused by an obstructive jejunal mesenteric hernia. There were no deaths related to IH overall.

## Discussion

The major findings of this observational cohort study, which included 785 consecutive patients with linear LRYGB in which 493 patients did not have mesenteric defects closed at the level of the EE and 292 received jejunal mesenteric defect closure during the primary operation, are as follows: (1) In our collective, the suture closure of the jejunal mesenteric defect leads to a reduction in absolute numbers of overall IH, but no statistically significant difference was found. (2) While IH appearance at the jejunal mesenteric space was reduced, a low rate of Petersen hernias in both groups was observed, as a T-shaped omental split was routinely performed in all patients.

The incidence of IH is a feared complication after LRYGB. According to the meta-analysis of Geubbels et al., the lowest rates of IH occur in antecolic gastric bypass with closure of both the jejunal mesenteric and the Petersen space [10]. Randomized controlled trials and large observational studies described IH rates slightly above 2% up to 5 years after surgery with closure of both gaps during primary surgery [13, 17]. In the current trial, reduction in IH rate was less pronounced. However, definition of IH differs widely in literature. In our collective, IH were clearly defined as manifest bowel herniation through the jejunal mesenteric or the Petersen space during revisional surgery. Open gaps without bowel herniation in symptomatic patients were assessed separately. Consequently, our IH rate might be underestimated, yet when considering all patients with open gaps and abdominal pain, intermittent IH cases would likely be overestimated given other causes of abdominal pain after LRYGB (e.g., symptomatic cholecystolithiasis). After approximately 3 years post-surgery, no more cases of IH occurred in the closure group. This disappearance of IH after mesenteric defect closure over time has been described in other collectives [17]. Unfortunately, our collective differs considerably in follow-up due to our change in closure strategy over time.

In our trial, we found that patients suffering from an IH or open mesenteric gap event had a lower BMI than patients without IH. Possibly, the higher weight loss associated with a loss of intra-abdominal fat mass leads to bigger mesenteric gaps, which may increase the risk for bowel herniation and strangulation.

Despite the advantages of reducing IH by closure of mesenteric defects, disadvantages caused by the closure technique itself have been described. In particular, early postoperative complications such as mesenteric hematoma and kinking are more frequently seen after closing mesenteric defects [13, 15]. However, Stenberg et al. showed that mesenteric defect closure can be performed safely, by focusing in particular on early postoperative small bowel obstruction by kinking of the EE [23].

In our cohort, we only observed very few early complications and kinking cases. This may be explained by our suture closure technique, which includes a fixation of the EE to the mesentery, preventing a high mobility of the anastomosis. Furthermore, we do not incise the mesentery at the level of the EE, and, consequently, the anastomosis does not drop below the level of the transverse colon and cannot move in all three dimensions. This leads to a stabilization of the EE, which may also explain the low rate of IH at the Petersen space in addition to the adhesions caused by the consequent T-shaped split of the omentum even in cases without massive central obesity. Assuming that most of our patients have an open gap behind the AL, the fixed EE blocks the bowel from herniating from the left to the right side of the abdominal cavity. The relatively high rate of IH at the level of the EE may also be explained by a learning curve of the suture closing technique. According to our current strategy, we take great care to close the defect precisely with small gaps between the stitches and suture up to the EE, including some sero-serosal stitches at the level of the EE.

Overall, the benefits of suturing the jejunal mesenteric space with a non-absorbable barbed thread during primary LRYGB were not as distinct in our collective as in other collectives [10, 12, 24].

This contribution underlines once more that even with primary closure of mesenteric defects, IH after LRYGB remains a problem of high priority, and exploratory laparoscopy should be performed in cases of suspicion of IH, no matter if the defects were closed during primary procedure or direct signs in computed tomography are not present.

### Limitations

This study has several limitations. Firstly, patients were not randomly assigned to both study arms, but we present a retrospective analysis of two cohorts before and after a change of treatment strategy. Therefore, follow-up time varies strongly amongst both study arms examined. In addition, the effect of the learning curve of the individual surgeons, especially for the closure technique of the jejunal mesenteric space, cannot be assessed utilizing our set of data. Moreover, data on IH was assessed from clinical follow-up data, and therefore, no routine, extra patient interviews, or questionnaires were carried out to explore possible IH symptoms. Finally, even though we assessed patients who received emergency surgery outside of our department, some cases of IH operated elsewhere could be missed in our assessment. Nevertheless, due to our long follow-up time, the good adherence of our patients to the clinic and the reliable communication system between bariatric surgery centers in our country, we estimate that we covered most of the postoperative late complications.

## Conclusion

The closure of the jejunal mesenteric defect by non-absorbable suture may reduce the rate of IH at the jejunal mesenteric space after LRYGB. However, the beneficial effect in our collective is smaller than expected, particularly in patients with good weight loss. Petersen IH rate remained low by consequent T-shape split of the omentum without suturing of the defect.

## References

[1] Arterburn DE, Olsen MK, Smith VA, Livingston EH, Van Scoyoc L, Yancy WS, Eid G, Weidenbacher H, Maciejewski ML (2015). Association between bariatric surgery and long-term survival. JAMA.

[2] Schauer PR, Burguera B, Ikramuddin S (2003). Effect of laparoscopic Roux-en Y gastric bypass on type 2 diabetes mellitus. Ann Surg.

[3] Schauer PR, Bhatt DL, Kirwan JP (2017). Bariatric surgery versus intensive medical therapy for diabetes - 5-year outcomes. N Engl J Med.

[4] Aminian A, Zajichek A, Arterburn DE, Wolski KE, Brethauer SA, Schauer PR, Kattan MW, Nissen SE (2019) Association of metabolic surgery with major adverse cardiovascular outcomes in patients with type 2 diabetes and obesity. In: JAMA - J. Am. Med. Assoc. American Medical Association (AMA), pp 1271–1282 10.1001/jama.2019.14231 PMC6724187 31475297

[5] Sjöström L, Peltonen M, Jacobson P (2014). Association of bariatric surgery with long-term remission of type 2 diabetes and with microvascular and macrovascular complications. JAMA - J Am Med Assoc.

[6] Wittgrove AC, Clark GW, Schubert KR (1996). Laparoscopic gastric bypass, Roux en-Y: technique and results in 75 patients with 3-30 months follow-up. Obes Surg.

[7] Lönroth H, Dalenbäck J, Haglind E, Lundell L (1996). Laparoscopic gastric bypass. Another option in bariatric surgery. Surg Endosc.

[8] Angrisani L, Santonicola A, Iovino P, Formisano G, Buchwald H, Scopinaro N (2015). Bariatric surgery worldwide 2013. Obes Surg.

[9] Angrisani L, Santonicola A, Iovino P, Vitiello A, Higa K, Himpens J, Buchwald H, Scopinaro N (2018). IFSO worldwide survey 2016: primary, endoluminal, and revisional procedures. Obes Surg.

[10] Geubbels N, Lijftogt N, Fiocco M, Van Leersum NJ, Wouters MWJM, De Brauw LM (2015). Meta-analysis of internal herniation after gastric bypass surgery. Br J Surg.

[11] Abasbassi M, Pottel H, Deylgat B, Vansteenkiste F, Van Rooy F, Devriendt D, D’Hondt M (2011). Small bowel obstruction after antecolic antegastric laparoscopic Roux-en-Y gastric bypass without division of small bowel mesentery: a single-centre, 7-year review. Obes Surg.

[12] Higa K, Ho T, Tercero F, Yunus T, Boone KB (2011). Laparoscopic Roux-en-Y gastric bypass: 10-year follow-up. Surg Obes Relat Dis.

[13] Stenberg E, Szabo E, Ågren G, Ottosson J, Marsk R, Lönroth H, Boman L, Magnuson A, Thorell A, Näslund I (2016). Closure of mesenteric defects in laparoscopic gastric bypass: a multicentre, randomised, parallel, open-label trial. Lancet.

[14] Amor IB, Kassir R, Debs T, Aldeghaither S, Petrucciani N, Nunziante M, Baqué P, Almunifi A, Gugenheim J (2019). Impact of mesenteric defect closure during laparoscopic Roux-en-Y gastric bypass (LRYGB): a retrospective study for a total of 2093 LRYGB. Obes Surg.

[15] Kristensen SD, Floyd AK, Naver L, Jess P (2015). Does the closure of mesenteric defects during laparoscopic gastric bypass surgery cause complications?. Surg Obes Relat Dis.

[16] Stenberg E, Ottosson J, Szabo E, Näslund I (2019). Comparing techniques for mesenteric defects closure in laparoscopic gastric bypass surgery—a register-based cohort study. Obes Surg.

[17] Aghajani E, Nergaard BJ, Leifson BG, Hedenbro J, Gislason H (2017). The mesenteric defects in laparoscopic Roux-en-Y gastric bypass: 5 years follow-up of non-closure versus closure using the stapler technique. Surg Endosc.

[18] Aghajani E, Jacobsen HJ, Nergaard BJ, Hedenbro JL, Leifson BG, Gislason H (2012). Internal hernia after gastric bypass: a new and simplified technique for laparoscopic primary closure of the mesenteric defects. J Gastrointest Surg.

[19] Blockhuys M, Gypen B, Heyman S, Valk J, van Sprundel F, Hendrickx L (2019). Internal hernia after laparoscopic gastric bypass: effect of closure of the Petersen defect—single-center study. Obes Surg.

[20] Schneider R, Gass JM, Kern B, Peters T, Slawik M, Gebhart M, Peterli R (2016). Linear compared to circular stapler anastomosis in laparoscopic Roux-en-Y gastric bypass leads to comparable weight loss with fewer complications: a matched pair study. Langenbeck's Arch Surg.

[21] SMOB.ch-Start. http://smob.ch/de/. Accessed 12 Aug 2020

[22] Prism-GraphPad. https://www.graphpad.com/scientific-software/prism/. Accessed 15 Apr 2020

[23] Stenberg E, Näslund I, Szabo E, Ottosson J (2018). Impact of mesenteric defect closure technique on complications after gastric bypass. Langenbeck's Arch Surg.

[24] Chowbey P, Baijal M, Kantharia NS, Khullar R, Sharma A, Soni V (2016). Mesenteric defect closure decreases the incidence of internal hernias following laparoscopic Roux-En-Y gastric bypass: a retrospective cohort study. Obes Surg.

